# Ex Vivo Human Foreskin Tissue Circumcision via High-Frequency Electric Welding

**DOI:** 10.3390/bioengineering13040411

**Published:** 2026-03-31

**Authors:** Xin Chen, Cai-Hui Zhu, Zhong-Zhen Hu, Cheng Liu, Ze-Wen Dong, Jian Qiu, Hui Zhao, Yang Li, Kai Fang, Si-Min Li, Jia-Kuan Liu, Dong Liu, Sheng-Jie Liang, Ke-Fu Liu, Chu-Hong Chen

**Affiliations:** 1 College of Intelligent Robotics and Advanced Manufacturing, Fudan University, Shanghai 200433, China; 2 Department of Urology, Shanghai Pudong Hospital, Fudan University Pudong Medical Center, 2800 Gong-Wei Road, Hui-Nan Town, Pudong, Shanghai 201399, China; 3 Shanghai Pudong Hospital, Fudan University Pudong Medical Center, 2800 Gong-Wei Road, Hui-Nan Town, Pudong, Shanghai 201399, China

**Keywords:** high frequency electric welding, heat damage analysis, stress test, surgical time

## Abstract

Background: Despite the clinical significance of circumcision, traditional suturing is frequently compromised by intraoperative bleeding and lengthy recovery periods. While high-frequency electric welding (HFEW) presents a compelling alternative, its utility in foreskin removal procedures remains unexplored. Methods: Employing freshly excised human foreskin tissues, this study simulated the circumcision procedure to benchmark HFEW against standard suturing techniques. Critical performance metrics, encompassing tensile integrity, thermal injury scope, and operative efficiency, were rigorously quantified. Results: HFEW demonstrated exceptional time efficiency, averaging 2.01 ± 0.9 min—a 77.02% reduction relative to conventional suturing (*p* < 0.001). However, mechanical testing revealed disparities in tissue adhesion; the HFEW cohort recorded lower forces for initial tearing (4.42 ± 1.02 N) and complete rupture (6.15 ± 1.65 N) compared to the superior tensile resistance of the suturing group (7.91 ± 3.26 N and 14.22 ± 6.91 N, respectively). Conclusions: Although HFEW yields comparatively lower tensile strength, its remarkable operational efficiency positions it as a viable technical innovation for circumcision. These preliminary findings support the pursuit of further in vivo investigations to confirm its clinical applicability.

## 1. Introduction

As a procedure with significant cultural and religious relevance, circumcision remains a key practice in modern medicine [1,2]. It is associated with a lower incidence of urinary tract infections, HIV, other sexually transmitted diseases, and penile cancers, making it vital for male health [3].

Circumcision methodologies are primarily classified as either traditional surgical techniques or device-assisted systems [4,5], with each presenting a unique profile of benefits, drawbacks, and technical intricacy [6]. The conventional “cut-and-suture” method involves the following steps [7]: using surgical forceps to mark the incision line, applying two additional clamps to the distal prepuce, incising the shaft skin at the coronal bulge to expose the coronal layer of the glans area, establishing a dissection plane lateral to the preputial epithelial layer to perform the circumcision, and finally, re-approximating the penile shaft skin just below the coronal sulcus using fine absorbable sutures. Despite its technical maturity, this method is gradually becoming obsolete due to modern techniques that offer advantages such as shorter operative duration and reduced blood loss [8]. Conversely, device-assisted systems improve efficiency but are limited by strict patient selection criteria and can compromise healing time [9,10]. These challenges are amplified in pediatric and elderly patients, who represent a substantial demographic for circumcision. They experience higher complication rates—including hemorrhage, infection, and balanitis—and demand greater consideration for technique adaptation, anesthesia options, and recovery management [11]. Therefore, an innovative approach that integrates the strengths of current technologies while mitigating their weaknesses is needed.

High-frequency electric welding (HFEW) offers distinct advantages over traditional methods, including shorter procedure times, reduced bleeding, no foreign bodies, and lower inflammation [12,13,14,15]. Its success in other surgical fields, such as thyroid and esophageal tumor resection, demonstrates its potential to overcome the deficiencies of conventional circumcision [16,17,18]. These advantages compensate for the deficiencies of conventional male circumcision, making the application of HFEW technology in this field feasible. However, a significant knowledge gap exists, as no empirical studies have investigated HFEW for human male circumcision.

However, the current difficulty in applying HFEW technology to circumcision lies in the lack of ex vivo experiments using human tissue and subsequent clinical trials. The advantages of using HFEW for ex vivo experiments are as follows: First, human tissue retains authentic anatomical layers, which is incomparable to animal models. Second, ex vivo experiments allow for “destructive” testing that is not feasible in clinical surgery. Furthermore, by strictly following standard clinical surgical steps during the ex vivo experiments, a comparative model between ex vivo circumcision and clinical surgical circumcision can be effectively established.

To bridge this critical void, our study represents a pioneering effort, conducting the first-ever ex vivo experiments on fresh human foreskin tissue. The primary objective is to establish the fundamental feasibility of HFEW in this specific surgical context. In this study, we innovatively designed a tensile test tailored to the histological structure of the foreskin, introducing a tensile strength metric to evaluate the integrity of HFEW incisions. Furthermore, thermal damage analysis and time comparisons were conducted to substantiate the potential application of HFEW. We anticipate that this foundational research will not only furnish a robust scientific basis for the future clinical translation of this technology but also pave the way for introducing a safer and more efficacious therapeutic alternative for patients.

## 2. Materials and Methods

### 2.1. Ethics Approval

The male preputial tissue for this study was sourced from Shanghai Pudong Hospital, Fudan University Pudong Medical Center. All experimental procedures adhered to the guidelines for the care and use of human clinical subjects and received approval from the Hospital’s Ethics Committee (Approval No. 2025-R-S-044).

### 2.2. Samples

**Figure 1:**
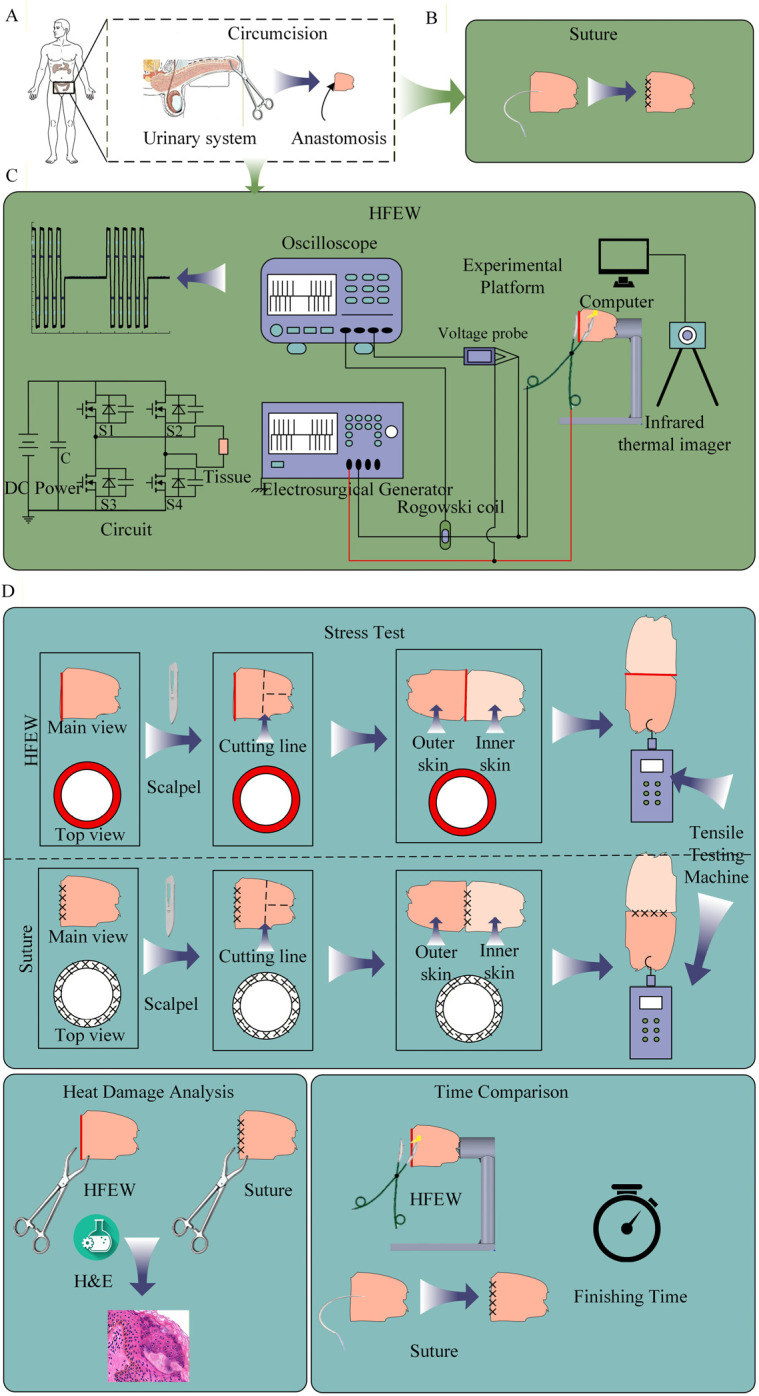
Experimental setups and surgical procedures. (**A**) Collection of Human Foreskin Tissue. (**B**) Suturing procedure. (**C**) HFEW procedure. (**D**) Procedures of stress test, heat damage analysis and time comparison between suturing and HEFW.

In this study, prepuce samples (*n* = 20) were randomly and equally divided into two groups. The samples were processed using a High-frequency electric welding (HFEW) technique and traditional cutting and suturing methods, respectively. Subsequently, a tensiometer was employed to evaluate the tensile strength of HFEW in circumcision. To preserve the biological activity of the male preputial tissue, all samples were processed for ex vivo experiments immediately following surgical resection. The male preputial tissues were randomly divided into the HFEW group and the Suturing group (Figure 1B,C).

### 2.3. Equipment

**Figure 2:**
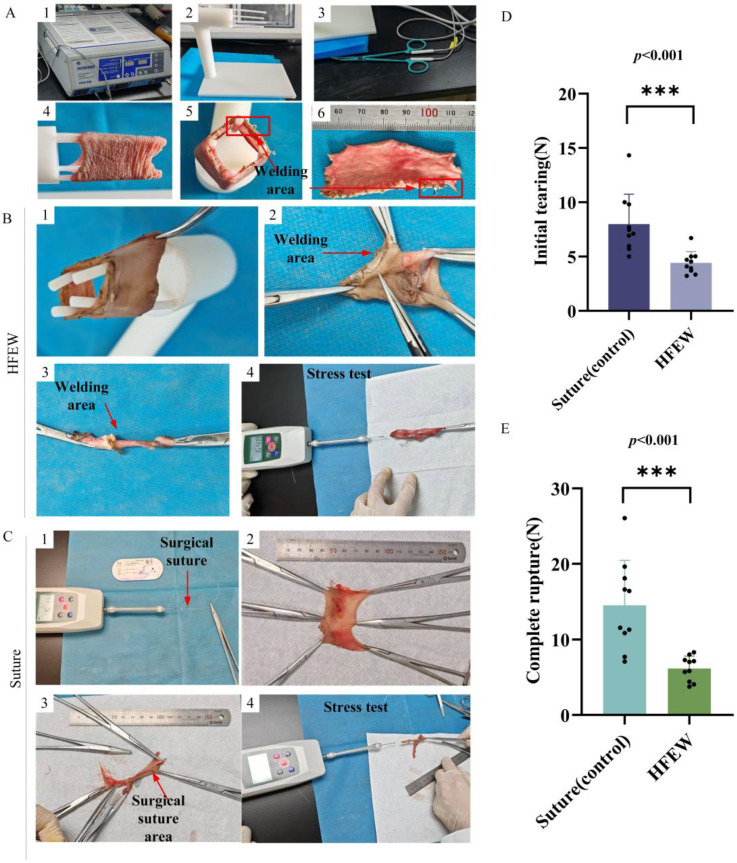
Breaking strength comparison between suture and HFEW. (**A**) Experimental set-up. (**B**) Steps of HFEW. (**C**) Steps of suture. (**D**) Starting breaking strength of suture and HFEW. (**E**) Ending breaking strength of suture and HFEW.

The equipment used in this study was a high-frequency electrosurgical generator, model EKVZ-300 (PATONMED, Kyiv, Ukraine) (Figure 2A), from the Institute of the National Academy of Sciences of Kyiv, Ukraine. Previous studies have shown that the EKVZ-300 can accommodate high-frequency electric field welding in various scenarios by altering the duty cycle and amplitude of the output voltage waveform [19]. Based on the voltage and current waveforms provided in the literature, the power consumption of the EKVZ-300 under different welding modes was calculated to be 100 W, 120 W, and 140 W, respectively. The high-frequency electrosurgical generator generated high-frequency pulse (with a bipolar voltage of 140, 180, 210 V, high voltage of 70, 90, 110 V, frequency of 390 kHz, and a 50% duty cycle) to cut the foreskin with welding forceps.

### 2.4. Experiment Procedure

All experiments were performed ex vivo using male preputial tissue obtained from patients immediately after circumcision. The tissue samples were subsequently disinfected. Similarly to surgical scissors, the welding forceps operated by automatically closing the wound. The width of the circumcision was between 1.0 cm and 1.5 cm.

The procedure for ex vivo circumcision using traditional needle-and-suture technique was as follows: the incisions on fresh human foreskin tissue were sutured with surgical thread, adhering to standard surgical protocols to simulate a real surgical procedure, until the entire incision was closed. The procedure for ex vivo circumcision using HFEW was as follows: the incisions on fresh human foreskin tissue were clamped using electrosurgical forceps connected to an electrosurgical generator, with an anastomosis time of 5 s, until the entire incision was anastomosed (Figure 1B,C).

Since male preputial tissue is annular and cannot be directly subjected to tensile testing, the preputial tissues from both the high-frequency electric field welding group and the surgical suture group were cut. This was performed to position the welded and sutured sites in the center for the tensile test. A tensile tester was then used to perform a horizontal tensile test. The strength of the high-frequency electric field welding and the surgical sutures was evaluated by recording the readings of the tensiometer when gaps first appeared at the welded and sutured sites and when the ends were completely separated (Figure 1D).

### 2.5. Experiment Parameters

The circumcision’s operative time was the entire period required for the incision and closure of the prepuce. The temperature at the welding site was continuously monitored using an infrared thermometer during the HFEW process, with the highest temperature recorded being deemed the surgical temperature.

### 2.6. Pathological Examination

For pathological examination, specimens were prepared as follows: the preputial tissue was cut at a distance of 1.0 cm from the tip, fixed in a formalin-buffered solution, embedded in paraffin, and then sectioned and stained with Hematoxylin and Eosin (H&E). The slides were analyzed to assess tissue degeneration and pathological changes. A total of seven specimen groups were examined, comprising one control group (untreated male preputial tissue), three groups for the HFEW, and three groups for the suturing.

### 2.7. Statistical Analysis

Data are expressed as mean ± standard deviation (SD). Statistical significance was assessed using Student’s *t*-test with SPSS software (version 22.2), and a *p*-value < 0.05 was considered statistically significant.

## 3. Results

### 3.1. Stress Test Between High-Frequency Electric Welding (HFEW) and Suture

The evaluation parameters were two: initial tearing of HFEW and suture, complete rupture of HEFW and suture. The rationale for establishing this evaluation parameter is twofold. Firstly, the male preputial tissue is anatomically divided into an outer and an inner layer; following a circumcision procedure, suturing is required to approximate the outer and inner preputial layers at the distal end of the penis. Secondly, it is designed to simulate the clinical scenario of incisional bleeding and dehiscence that can occur during circumcision surgery. The power of the electrosurgical generator was 140 W. The preputial tissue was secured on the experimental platform (Figure 2(A2)). HFEW was then performed using electrosurgical forceps (Figure 2(A3)) connected to an electrosurgical generator (Figure 2(A1)), with the resulting thermal damage achieving the desired welding effect (Figure 2(A4,A5)).

Regarding tensile strength, the HFEW group exhibited a force for initial tearing of 4.42 ± 1.02 N and for complete rupture of 6.15 ± 1.65 N (Figure 2D,E). In comparison, the traditional technique group demonstrated a force for initial tearing of 7.91 ± 3.26 N and for complete rupture of 14.22 ± 6.91 N (Figure 2D,E).

Statistical analysis revealed that although the mean values for the HFEW group were lower than those of the traditional control group, the variance was significantly smaller, indicating superior stability in strength. It is important to note that the higher mean tensile values observed in this study are attributed to the fact that the measurement primarily reflects the strength of the suture material itself, rather than the true strength of the anastomosis. This discrepancy, which is inherent to the measurement method, introduces a systematic error. Future research should focus on refining the experimental methodology to reduce this error and achieve more precise measurements.

### 3.2. Heat Damage Analysis

Thermal injury is a significant adverse effect in electrosurgical procedures that warrants meticulous attention. In our experimental study, the high-frequency electric field welding group produced minimal smoke, and the edges of the welded site exhibited a yellowish eschar. Infrared thermography was employed to monitor the preputial tissue temperature throughout the welding process.

**Figure 3:**
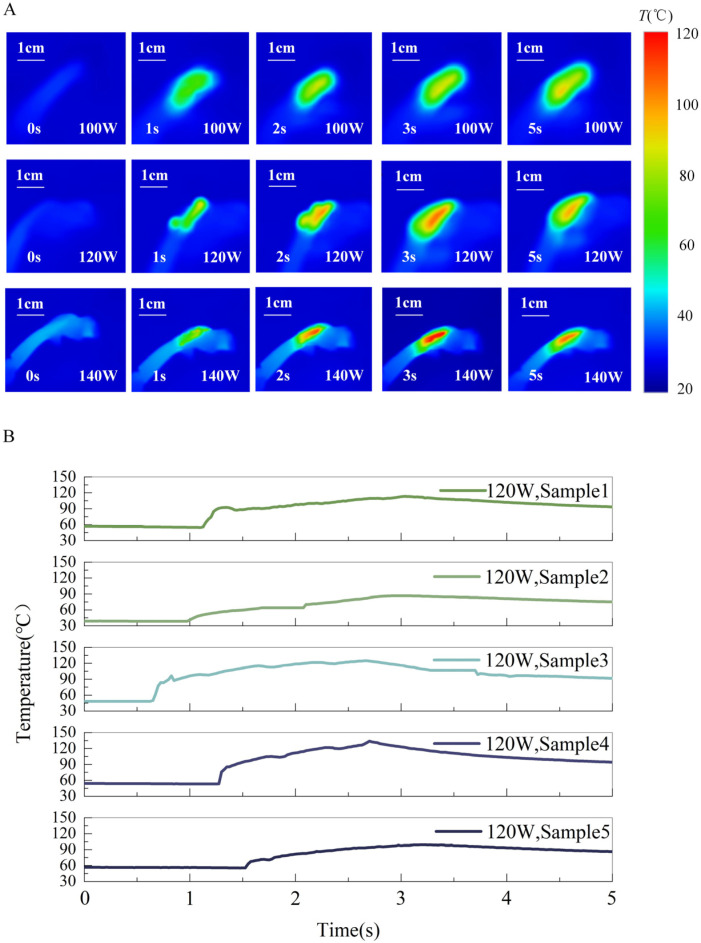
(**A**) Infrared thermography of HFEW in different power levels. (**B**) Measurements of transient temperature in preputial tissue during HFEW at the same power level.

Initially, a parametric study was conducted to determine optimal welding settings, utilizing three distinct power levels: 100 W, 120 W, and 140 W. All welding procedures were performed at an ambient temperature of 20 °C with a fixed duration of 5 s. The results revealed that under all three power settings, the epidermal temperature peaked at approximately 3 s, followed by a gradual decline. The highest temperature, reaching 120 °C, was recorded at the 140 W setting (Figure 3A).

Based on these preliminary findings and in conjunction with data from prior research, a power of 120 W was selected for subsequent experiments. Five additional samples were welded under this condition for instantaneous temperature monitoring. The peak instantaneous temperatures for these five samples ranged from 90 °C to 120 °C. This thermal output is substantially greater than that associated with conventional cutting and suturing techniques. These findings suggest that, in comparison to traditional methods, HFEW may induce a greater degree of thermal damage to the preputial tissue (Figure 3B).

**Figure 4:**
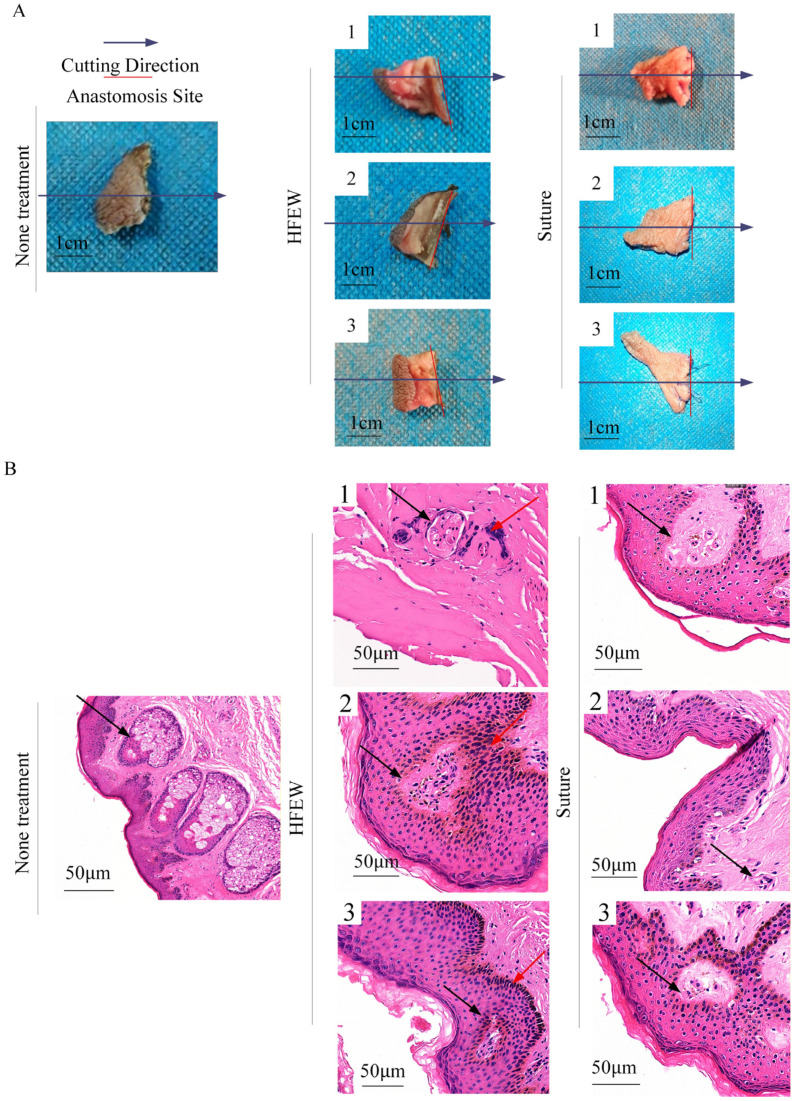
Pathological analysis of tissue thermal injury after circumcision using HFEW. (**A**) Tissue processing. (**B**) No treatment group, HFEW and suture group (red arrows: thermal injury area; black arrows: tissue cells).

Pathological analysis is a key indicator for assessing the degree of thermal injury. This research presents the pathological analysis conducted on seven distinct sample groups (Figure 4A). One group comprised a control group, which received no treatment, three groups treated with HFEW, and three groups subjected to conventional suture techniques. Quantitative analysis of the extent of coagulative necrosis revealed that the zone of thermal injury was less than 1 mm (Figure 4B), indicating that HFEW induces only limited thermal damage during circumcision. Furthermore, this technique was shown to be effective in vessel coagulation.

A more detailed histological examination demonstrates a clear contrast in vascular structures between normal preputial tissue and the areas of coagulative necrosis. While vasculature was readily identifiable in the normal tissue, it became indistinguishable within the necrotic zones, suggesting complete vessel occlusion. Higher-magnification observation of cellular morphology within the coagulative necrotic areas revealed tightly packed cells, some of which exhibited elongated shapes, damaged cell membranes, and vacuolated, flaky cytoplasm. A notable increase in eosinophilic staining was also ob-served. The observation of pyknosis, karyolysis, and karyorrhexis, resulting from the leakage of nuclear material, demonstrates that HFEW causes significant morphological changes and ultimately cell disruption.

### 3.3. Time Comparison Between HFEW and Suture

**Figure 5:**
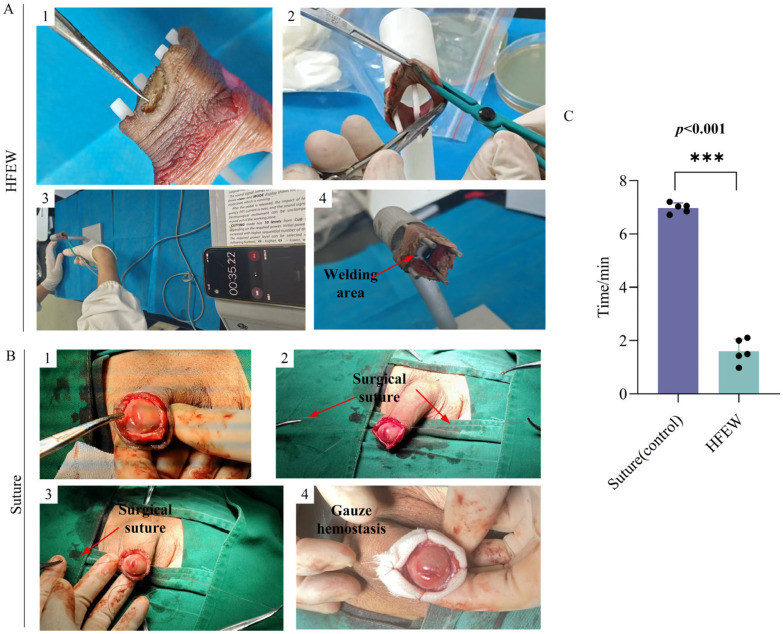
Experimental results and analysis of circumcision operative duration between HFEW and suture. (**A**) Experimental procedure of HFEW. (**B**) Experimental procedure of suture. (**C**) Statistical comparison of operative time between HFEW and suturing comparison.

HFEW represents an advanced surgical technique that integrates the functions of electrosurgical incision, electrocoagulation, and tissue welding into a single platform. This integration streamlines the surgical procedure and substantially reduces operative duration. In the present study, a comparative analysis was conducted between HFEW and traditional suturing, each performed on five groups of samples. The time required to complete a circumferential excision and closure of the prepuce was meticulously recorded. It is important to note that the suturing procedures were performed by a qualified urologist in an actual surgical setting (Figure 5B). The results demonstrated that the mean operative time for circumcision using the HFEW technique was merely 2.01 ± 0.9 min, which corresponds to only 22.98% of the time required for the conventional incision-suture method—a reduction of approximately 77.02% (Figure 5C). These findings underscore the significant advantage of HFEW in reducing operative time, thereby presenting a novel option for enhancing both surgical efficiency and patient safety.

## 4. Discussion

### 4.1. Advantages of High-Frequency Electric Welding (HFEW)

Circumcision in clinical practice can be executed using a range of techniques, including standard surgical dissection and device-assisted methods like the AlisClamp, ShangRing, PrePex, Unicirc, Mogen clamp, and Gomco clamp, among others [6,20,21,22,23]. Each modality has its own set of characteristics and benefits, notably concerning the duration of the procedure and the amount of blood lost during the operation. However, these are linked to some limitations, like lengthy recovery periods and complicated procedures.

HFEW has emerged as a novel technology in the surgical field. It has been shown to be effective in numerous surgical procedures, offering significant benefits such as reduced surgery time, less bleeding during the operation, and faster recovery post-surgery [16,17,24,25].

This study advances the field by conducting the first ex vivo experiments on fresh human foreskin tissue, substantiating HFEW’s advantages in reducing operative time and ensuring safety. These results provide a strong basis for future clinical trials.

The time taken for a surgical operation directly determines the anesthesia dosage required, thereby impacting patient safety [26,27]. Circumcision operative time varies by technique. While the traditional method averages 30 min and the Alislamp technique reduces this by 42.22%, HFEW drastically cuts the time to a mean of 2.01 ± 0.9 min. This represents a 77.02% reduction compared to the conventional method, positioning HFEW as a novel option for enhancing both surgical efficiency and patient safety.

### 4.2. Future Work

This study was limited to ex vivo experiments on human foreskin tissue, which confirmed the robustness and safety of HFEW through tensile strength and thermal damage analysis. Future research will transition to in vivo investigations.

Firstly, the design and optimization of the welding device are paramount. The current experimental platform is unsuitable for surgery, so the team plans to develop a new clinical stapler by integrating principles from existing circumcision devices with electrosurgery. This novel stapler will perform HFEW before cutting the tissue, an approach expected to maximize the technology’s advantages by achieving both rapid surgery and minimal blood loss.

Secondly, during surgical operations, blood loss poses a significant safety risk that requires careful control to prevent severe complications or fatalities [28,29]. As a result, measuring blood loss during surgery has become crucial for assessing the safety of surgical methods. Earlier research using conventional incision and suture methods for circumcision in animal models indicated an average blood loss of about 22.35 ± 5.17 mL. In modern medical practice, the Shang Ring and Mogen Clamp methods, which control bleeding by applying pressure, have greatly minimized bleeding during surgery, occasionally leading to procedures without any blood loss. Based on the application of HFEW in other anastomotic scenarios [11,12,13], the use of this high-frequency electric field welding technology can, achieve a zero-blood-loss effect during surgery. This is due to the method’s ability to use thermal effects to cut tissue and achieve electrocoagulative hemostasis at the same time. This finding demonstrates the pronounced advantage of high-frequency electric field welding in controlling hemorrhage, offering a safer and more efficacious option for surgical intervention. This critical parameter will be incorporated into the experimental design of future in vivo studies.

Lastly, postoperative recovery time is a pivotal parameter for evaluating surgical outcomes, although it could not be assessed in the current ex vivo study. Recovery time is a critical metric for gauging the efficacy of surgical methods. When comparing recovery times across different circumcision techniques, conventional procedures typically require an incision healing time of 16.50 ± 2.57 days. However, the potential for intraoperative bleeding may necessitate extended periods of observation and convalescence for patients. The study [30] observed canines undergoing circumcision with HFEW and found a significant reduction in recovery time by 25.27% compared to traditional incision and suture methods. This accelerated recovery is likely attributable to several factors, such as the absence of intraoperative bleeding and the lack of a foreign-body reaction to sutures. It is apparent that HFEW has a notable advantage in recovery time over other circumcision procedures, allowing patients a surgical option for a faster return to normal activities.

### 4.3. Applications Prospects

HFEW significantly improves circumcision by combining rapid tissue dissection with simultaneous vessel sealing. This dual action greatly reduces operative time and blood loss. By eliminating foreign implants, HFEW also lowers rejection risk and accelerates recovery. Its precision minimizes surgical trauma, thereby decreasing postoperative complications like infection, edema, and pain, while also creating neater, more esthetic incisional margins.

Despite its promise, widespread HFEW adoption is currently hindered by high costs, limited device availability, the need for specialized surgeon training, and variable patient acceptance. However, as next-generation electrosurgical units are developed and more clinical trial data emerges, HFEW is poised to become a safer, more effective, and more common therapeutic option for circumcision.

## 5. Conclusions

This study represents the first investigation to apply HFEW to ex vivo experiments using human foreskin tissue. A comprehensive comparison with conventional suture techniques was conducted, focusing on three critical parameters: burst strength, thermal damage, and procedural time. The results conclusively demonstrate that HFEW significantly reduces operative duration while achieving sufficient weld strength to support postoperative recovery. Furthermore, the technique offers the advantages of simplified operation and the elimination of foreign material implantation. Based on these compelling findings, this technology is validated as a promising candidate for future in vivo clinical trials in humans.

## Data Availability

The datasets generated during and/or analyzed during the current study are available from the corresponding author on reasonable request.
